# Combined effects of ocean acidification, warming, and salinity on the fertilization success in an Arctic population of sea urchins

**DOI:** 10.1038/s41598-025-27725-z

**Published:** 2025-12-18

**Authors:** Nadjejda Espinel-Velasco, Ane Cecilie Kvernvik, Haakon Hop, Sam Dupont

**Affiliations:** 1 Norwegian Polar Institute, Fram Centre, Tromsø, 9296 Norway; 2 Present Address: Department of Marine Science, Tjärnö Marine Laboratory, University of Gothenburg, Gothenburg, Sweden; 3 Department of Biological and Environmental Sciences, University of Gothenburg, Kristineberg, Gothenburg, Sweden; 4 Marine and Freshwater Research Institute, Fornubudir 4, Hafnarfjörður, 220 Iceland

**Keywords:** *Strongylocentrotus droebachiensis*, Global change related stressors, Anthropogenic pressures, Multiple stressors, Benthic communities, Early-life processes, Kongsfjorden, Svalbard, Ecophysiology, Marine biology, Environmental impact

## Abstract

Anthropogenic stressors, including ocean acidification (OA), ocean warming (OW), and salinity changes, are rapidly altering marine ecosystems, with Arctic regions being particularly vulnerable. This study investigates the combined effects of these stressors on the fertilization success of the green sea urchin (*Strongylocentrotus droebachiensis*) from Kongsfjorden, Svalbard. We exposed gametes to various levels of pH, temperature, and salinity to assess their individual and combined impacts on fertilization performance. Our results show that temperature and pH significantly influenced fertilization success, with temperature having the strongest effect, while salinity had no significant impact. A significant statistical interaction between temperature and pH indicated that warming enhanced fertilization more effectively at higher pH levels, while low pH suppressed this increase. To compare the relative influence of each stressor, we used a conceptual model based on standardized slopes, which supported temperature as the dominant driver, followed by pH. These findings highlight the importance of considering the effects of combined stressors when assessing marine organism responses to climate change, especially in polar ecosystems. Our study underscores the need for further research into the mechanisms driving these combined effects, given that Arctic ecosystems face accelerated environmental changes.

**Supplementary Information:**

The online version contains supplementary material available at 10.1038/s41598-025-27725-z.

## Introduction

Anthropogenic stressors such as ocean acidification (OA) and ocean warming (OW) are negatively impacting marine ecosystems worldwide^1^. Polar ecosystems, and particularly Arctic ecosystems, are among the most sensitive ecosystems to these stressors^2–4^. Arctic marine ecosystems are experiencing some of the fastest rates of warming and acidification globally, along with a continued decline in sea-ice volume^5^. These adverse effects are being exacerbated by additional stressors already present in coastal ecosystems, such as pollution or freshening^6^.

Due to their sensitivity to environmental stressors, early-life stages and their associated processes (e.g. fertilization, metamorphosis) are considered critical bottlenecks in the life cycle of marine benthic organisms^7^. Understanding how future environmental changes will impact them is therefore crucial. Extensive research has been conducted on fertilization success in broadcast spawners under future scenarios of global change, particularly focusing on acidification conditions (reviewed in^8^). The findings suggest that sea urchin fertilization is robust to small changes in pH^9–12^. However, potential negative impacts are anticipated at more extreme levels^13^.

The bulk of this research has concentrated on tropical and temperate species, leaving polar species – especially Arctic ones – relatively understudied. Furthermore, the majority of Arctic research has focused on single-stressor responses such as OA, while it has become evident that a multiple-stressor approach is more suitable to uncover complex combined effects^14^. Multiple-stressor studies are therefore essential to fully understand the extent to which future scenarios of global change will impact marine ecosystems.

Future environmental changes in the Arctic are expected to alter the dynamics of benthic and coastal communities, particularly around Svalbard. Understanding how these communities will fare is vital for informing management practices and policy development. In this context, we investigated the effects of pH, temperature and salinity on the fertilization success of the green sea urchin (*Strongylocentrotus droebachiensis*) collected from Kongsfjorden, a glacial fjord in the Svalbard archipelago. This species has a circumboreal distribution, extending from temperate to Arctic regions, and plays a central role in the benthic ecosystem of Kongsfjorden^15^. The population in this fjord seems to be increasing, particularly in the outer reaches, where sea urchins have grazed down significant portions of kelp biomass^16^. Known for its utility in developmental and environmental biology, *S. droebachiensis* is well-suited for laboratory studies dues to its ease of collection, maintenance, and spawning.

Kongsfjorden is one of the most extensively studied Arctic fjord systems and serves as a reference site for marine science and monitoring^17^. The unique diversity and abundance of its fauna make it an important early indicator of changes associated to global-change stressors, such as acidification or warming^17^. Furthermore, ongoing monitoring efforts in the area document temporal changes in environmental parameters (e.g.^18,19^).

In this study, we examined the fertilization of *S. droebachiensis* gametes under current and extreme levels of seawater pH, temperature, and salinity. Additionally, we assessed the relative contributions of each stressor by developing an index based on performance curves for individual stressors, aiming to discern the mechanisms underlying the influence of these environmental stressors on fertilization in this sea urchin population.

## Materials and methods

### Animal collection and acclimation

**Fig. 1:**
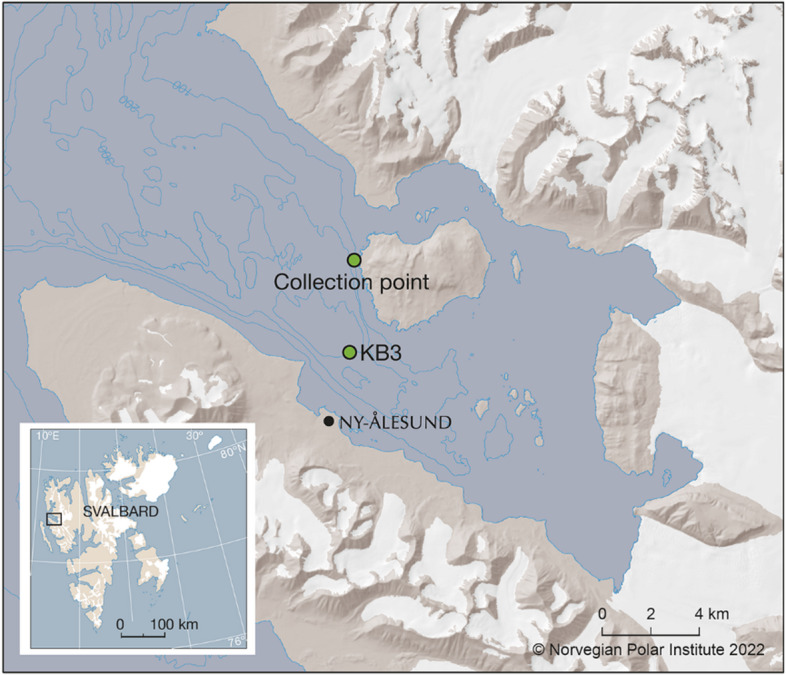
Location of the sea urchin collection site in Kongsfjorden (diving site at Hansneset, Blomstrand), Ny-Ålesund with location of the marine laboratory (experimental site), and the Kb3 station (used for reference environmental data; Fransson et al., 2016). Figure created with ArcMap 10.8.2. and Adobe Illustrator 2022. Map basis and cartography by the Norwegian Polar Institute, hydrographic data from the Norwegian Mapping Authority.

Adult green sea urchins were collected in June 2021 by scuba divers from Kongsfjorden, Svalbard (78°59’05.0”N, 11°57’55.0”E; Fig. 1) at depths of 3 to 10 m. Although biometric measurements were not taken, all individuals were sexually mature, as indicated by the presence of ripe gametes and successful spawning following KCl injection. The urchins were transported to the Ny-Ålesund marine laboratory (Ny-Ålesund, Svalbard) acclimated in flow-through tanks supplied with ambient Kongsfjorden seawater (temperature ~ 2 °C, salinity ~ 34, and pH ~ 8.1) and fed *ad libitum* with seaweed for one week before the experiments commenced.

### Fertilization assays and experimental design

Spawning was induced by means of intracoelomic injection of 0.1 M KCl and gametes were collected separately for quality check. Eggs were collected in glass vials with filtered seawater and inspected for viability (shape and colour). Sperm was collected dry and kept on ice, and quickly inspected for viability (activation when in contact with seawater). Gametes of two females and four males were selected and pooled prior to fertilization assays to reduce individual variability and represent the population-level response.

A pilot fertilization assay (five concentrations, four replicates) was carried out to establish the sperm concentration that would lead to a fertilization success of approximately 50% to optimize the chances of observing effects^20^. Details and results of this pilot study can be found in supplementary material (S1).

The main fertilization assays took place in small 24 multi-well plates and involved testing four pH levels (nominal pH 7.1, 7.4, 7.7 and 8.1), four salinities (28, 30, 32 and 34) and three temperatures (1.5 °C, 2.2 °C and 5.3 °C). The selected levels of pH, salinity, and temperature were chosen to reflect both current and projected variability in Kongsfjorden. Reference conditions (pH 8.1, salinity 34, temperature 2.2 °C) correspond to average field measurements^18^, while altered conditions represent extreme but plausible future scenarios under ongoing climate change^19^. The experimental design included a factorial combination of the three temperatures and four salinities at pH ~ 8.1, as well as a factorial combination of the same three temperatures and four pHs at ambient salinity of 34, resulting in a total of 21 treatments. Each treatment was replicated six times, and the controls were replicated 12 times, adding up to a total of 144 experimental units (Table 1).

**Table 1:** Treatments used for the fertilization assay.

Treatment	pH	Salinity	Temperature	Replicates	Treatment	pH	Salinity	Temperature	Replicates
T1	8.1	28	1.5	6	T11	8.1	34	2.2	12
T2	8.1	28	2.2	6	T12	8.1	34	5.3	12
T3	8.1	28	5.3	6	T13	7.7	34	1.5	6
T4	8.1	30	1.5	6	T14	7.7	34	2.2	6
T5	8.1	30	2.2	6	T15	7.7	34	5.3	6
T6	8.1	30	5.3	6	T16	7.4	34	1.5	6
T7	8.1	32	1.5	6	T17	7.4	34	2.2	6
T8	8.1	32	2.2	6	T18	7.4	34	5.3	6
T9	8.1	32	5.3	6	T19	7.1	34	1.5	6
T10	8.1	34	1.5	12	T20	7.1	34	2.2	6
					T21	7.1	34	5.3	6

For the assay, each individual well was filled with 2 ml of filtered seawater (FSW, 0.2 μm) from the corresponding treatment. Subsequently, approximately 200 eggs were added and left to sink to the bottom of the well. After 5 min, 1 µl of sperm dilution (at a concentration of 3.9 × 10^6^ sperm/ml) was added in each well and the plates were closed and incubated in a temperature-controlled water bath at the target temperature for 15 min. After that time, one drop of 4% paraformaldehyde (PFA) was added to each well to stop the fertilization process and fix the eggs. The fertilization success in each well was subsequently assessed under a microscope, with the appearance of the perivitelline membrane indicating successful fertilization (see supporting data in^21^).

### Preparation of treatments and measurements

The pH treatments were prepared by bubbling pure CO_2_ into FSW and mixing manually until reaching target values. For pH, the target values were 7.1, 7.4, 7.7 and 8.1. The pH measurements were taken manually with a pH probe (Hanna Instruments, model HI-98190) calibrated with NIST buffers (LabChem, Zelie-nople, PA, USA), and the final pH values for the treatments were pH_NBS_ 8.04 ± 0.01, pH_NBS_ 7.62 ± 0.04, pH_NBS_ 7.36 ± 0.06 and pH_NBS_ 7.14 ± 0.04. The salinity treatments were prepared in advance by diluting FSW with tap water, until reaching four final salinities: 28, 30, 32 and 34. The temperature conditions were achieved and maintained by means of water baths inside controlled temperature rooms (5.28 °C ± 0.02, 2.18 °C ± 0.05 and 1.55 °C ± 0.06). All physical parameters for the different treatments were measured and recorded immediately before the start of each assay (Table 2). The carbonate chemistry parameters of the experimental water were calculated using the CO2sys package^22^ and are reported in Table 2, using as input the pH measurements of the experimental water and values of total alkalinity (TA) obtained from^18^ for Kongsfjorden.

**Table 2:** Mean seawater parameters in the different treatments during the fertilization experiment.

Treatment	S	T(℃)	pH_NIST_	TA (mmol/kg)	fCO2 (matm)	pCO2 (matm)	HCO_3_ (mmol/kg)	CO_3_ (mmol/kg)	Ωcalc.	Ωarag.
T1	28	1.60	8.04	2228.00	398.87	400.59	2024.91	81.55	2.02	1.24
T2	28	2.20	8.06	2228.00	380.35	381.98	2011.72	86.94	2.15	1.33
T3	28	5.20	8.03	2228.00	415.72	417.43	2002.14	91.20	2.25	1.40
T4	30	1.60	8.04	2228.00	392.43	394.13	2013.26	85.41	2.09	1.30
T5	30	2.20	8.06	2228.00	374.06	375.66	1999.40	91.03	2.23	1.39
T6	30	5.20	8.03	2228.00	408.69	410.37	1989.37	95.46	2.34	1.46
T7	32	1.60	8.04	2228.00	386.89	388.56	2001.53	89.27	2.17	1.36
T8	32	2.20	8.06	2228.00	368.63	370.21	1987.01	95.10	2.31	1.44
T9	32	5.20	8.03	2228.00	402.59	404.25	1976.53	99.70	2.42	1.52
T10	34	1.60	8.04	2228.00	382.22	383.87	1989.76	93.10	2.24	1.41
T11	34	2.20	8.06	2228.00	364.03	365.59	1974.58	99.15	2.38	1.50
T12	34	5.20	8.03	2228.00	397.41	399.05	1963.66	103.91	2.49	1.57
T13	34	1.50	7.65	2228.00	1000.98	1005.31	2124.59	40.33	0.97	0.61
T14	34	2.10	7.67	2228.00	957.49	961.60	2117.52	43.14	1.04	0.65
T15	34	5.30	7.55	2228.00	1304.34	1309.70	2132.43	37.52	0.90	0.57
T16	34	1.40	7.41	2228.00	1773.18	1780.86	2167.52	23.58	0.57	0.36
T17	34	2.10	7.42	2228.00	1740.45	1747.91	2164.48	24.80	0.60	0.37
T18	34	5.30	7.24	2228.00	2722.73	2733.92	2180.16	18.79	0.45	0.28
T19	34	1.70	7.15	2228.00	3274.21	3288.34	2193.99	13.28	0.32	0.20
T20	34	2.30	7.19	2228.00	2995.32	3008.14	2189.89	14.89	0.36	0.23
T21	34	5.30	7.07	2228.00	4055.49	4072.16	2195.47	12.79	0.31	0.19

Salinity, temperature and pH were directly measured. Total alkalinity (TA) values were obtained from^18^, and remaining carbonate chemistry parameters were calculated using the CO2SYS software.

### Statistical analyses

All statistical analyses were performed with R v. 4.0.3. and RStudio v.1.4.1103^23^. A linear model (package “stats”) was used to test for effects of pH, temperature, salinity and their interactions on the fertilization success. The model and final data obtained are reported in the results section. The exploratory models and validation plots are detailed in Supplementary 2.

### Calculation of relative contributions and stress index

To quantify the relative contribution of each driver to fertilization success, we fitted simple linear regressions for each individual variable (pH, salinity, and temperature), using subsets of the data where the other two variables were held constant at reference levels (pH 8.1, temperature 2.2 °C, and salinity 34; see Supplementary 3). These reference conditions were chosen to represent typical environmental values at Kb3, the closest oceanographic monitoring station to our sea urchin sampling site in Kongsfjorden^18^ (Fig. 1).

The slope of each regression was used to calculate a comparative index of contribution, standardized relative to a one-unit change in pH (Supplementary 3 and supporting data in^24^). Specifically, the contribution of temperature (CT) relative to pH was calculated as the ratio between the slope of the pH model and the slope of the temperature model (slope_pH / slope_temp). Similarly, the contribution of salinity (CS) was derived from the ratio between the slope of the pH model and the slope of the salinity model (slope_pH / slope_sal). This approach allowed us to express each stressor’s contribution in standardized units for conceptual comparison. The relative stress index for each variable was calculated individually and used as input for the total stress index (TS). In this context, ‘stress’ is defined as the departure from reference conditions for a given environmental driver (e.g., deviation from pH 8.1), weighted by the relative effect size (slope) estimated from the simple linear models. The stress related to pH (S_PH_) was calculated by subtracting the reference pH value (pH_REF_ = 8.1) from the pH of each observation (pH_O_). The stress related to temperature (S_T_) was calculated by subtracting the reference temperature value (T_REF_ = 2.2°C) from the temperature for each observation and dividing the result by the calculated contribution value for the temperature, according to the following formula:

S_T_ = [(T_O_ – T_REF_)/ C_T_

Where: S_T_ = stress related to temperature, T_O_ = temperature in each observation,

T_REF_ = reference temperature (2.2 °C) and C_T_ = contribution due to the temperature.

The salinity-related stress was calculated by subtracting the reference salinity value (S_REF_ = 34) from the salinity for each observation and dividing the result by the calculated contribution value for the salinity, according to the following formula:

S_S_ = - [(S_O_ – S_REF_)/ C_S_

Where: S_S_ = stress related to salinity, S_O_ = salinity in each observation, S_REF_ = reference salinity (34) and C_S_ = contribution due to the salinity.

Finally, the total stress (TS) was calculated additively, according to the following formula:

TS = S_pH_ + S_T_ + S_S_.

Where: TS = Total stress; S_pH_ = stress related to pH; S_T_ = Stress related to temperature and S_S_ = stress related to salinity.

The fitted regressions and subsequent calculations are reported in the result section (Fig. 3), the model details and verification plots are in Supplementary 3.

## Results

### Effects of temperature, salinity and pH on the fertilization success

**Fig. 2:**
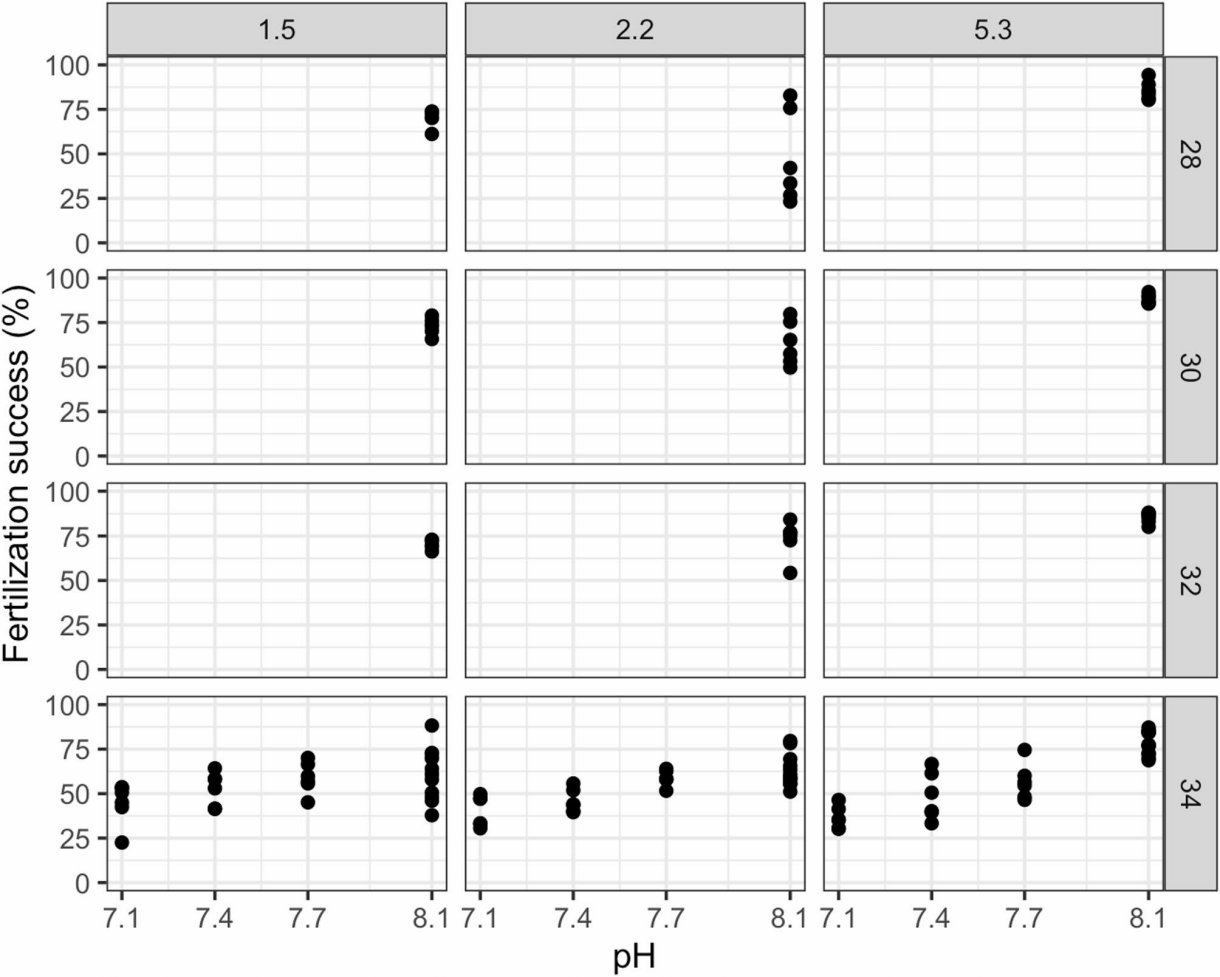
Fertilization success (%) per temperature (vertical facets), salinity (horizontal facets) and pH (x-axis).

Fertilization rates across all treatments ranged from 22.5% (at pH 7.1, salinity 34 and temperature 1.5 °C) to 94.3% (at pH 8.1, salinity 28 and temperature 5.3 °C; Fig. 2; and supplementary data in^21^).

The analysis revealed that fertilization success was significantly influenced by pH (negative effect) and temperature (positive effect), as well as their interactions (*p* < 0.05 and *p* < 0.001, respectively, Fig. 2, Supplementary 2). In contrast, salinity alone and its interaction with temperature did not significantly affect fertilization (*p* = 0.96 and *p* = 0.52, respectively, Fig. 2, Supplementary 2).

### Relative contribution of environmental drivers to the fertilization success

**Fig. 3:**
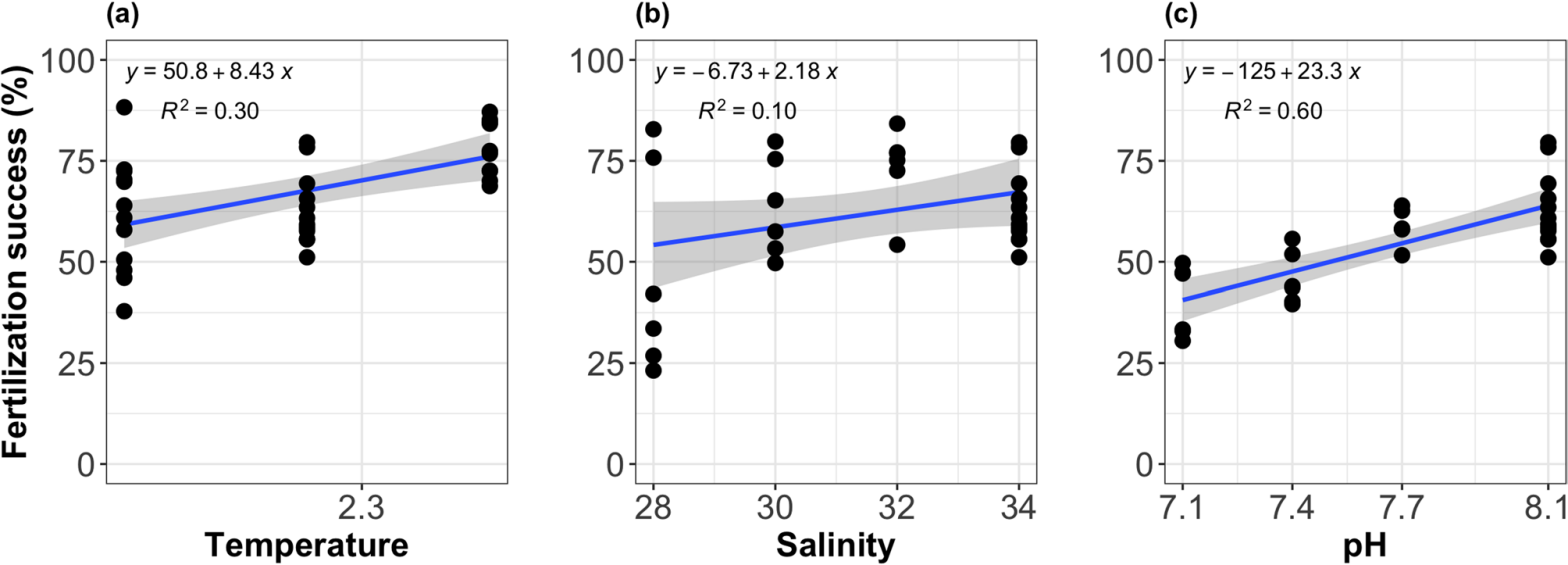
Fertilization success (%) at the reference conditions according to (**a**) temperature, (**b**) salinity and (**c**) pH.

At reference conditions of pH 8.1 and salinity 34 (Fig. 3a), fertilization success at the three tested temperatures ranged from 37.9% to 88.2% at 1.5 °C, which was similar but less than the upper value at 5.3 °C. At the reference conditions of pH 8.1 and temperature 2.2 °C (Fig. 3b), fertilization rates across different salinities varied from 23.2% (at salinity 28) to 84.2% (at salinity 32). Under reference salinity (34) and temperature (2.2 °C), fertilization success increased with pH, ranging from 30.5% (at pH 7.1) to 79.6% (at pH 8.1) (Fig. 3c). These modelled relationships are derived from simple linear regression models fitted separately for each driver under fixed reference conditions. They are intended to illustrate the relative influence of temperature, salinity, and pH on fertilization under standardized scenarios (Fig. 3). Full model coefficients and diagnostics are provided in Supplementary 3.

**Fig. 4:**
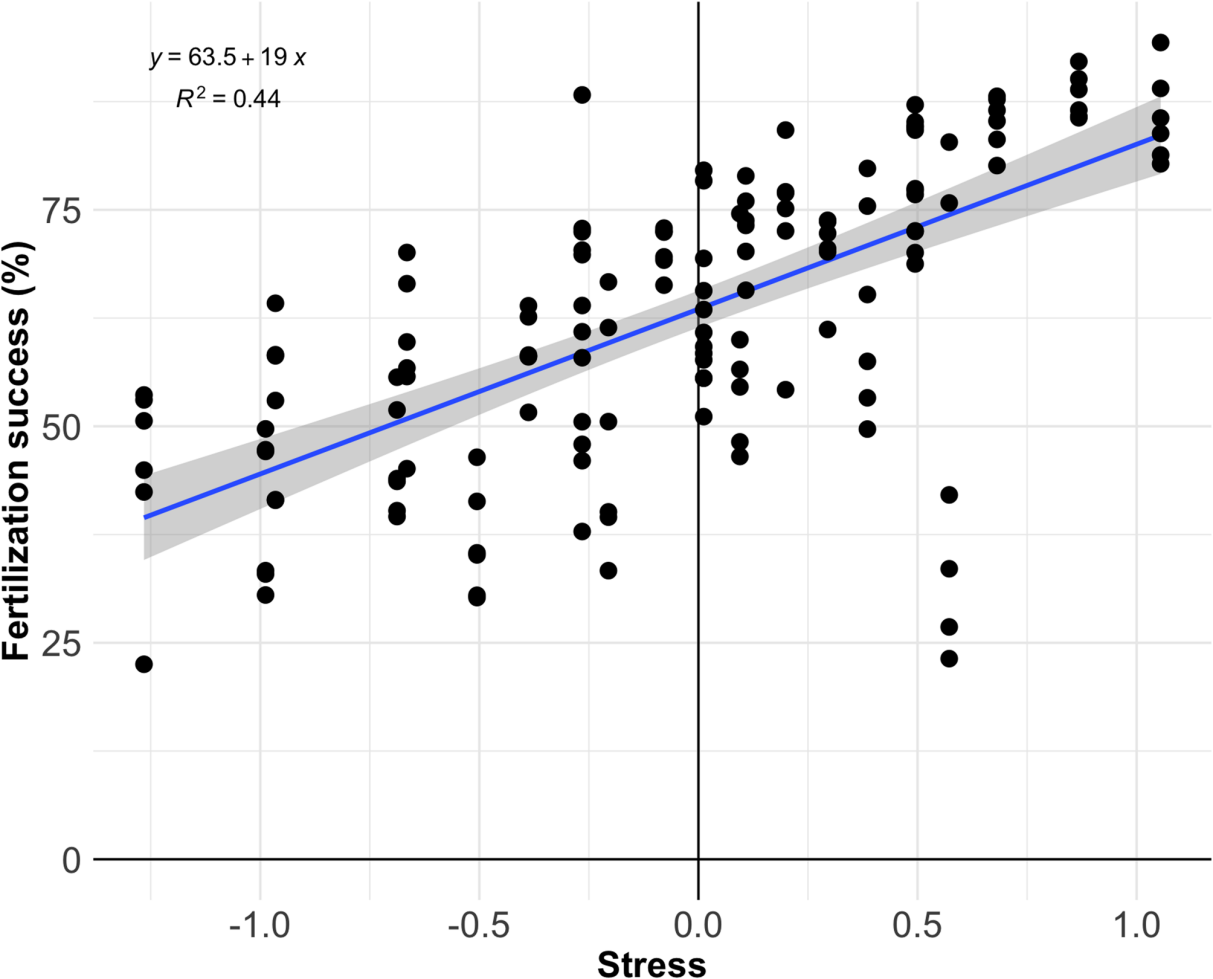
Calculated stress (CS) index based on the relative contribution of all environmental variables.

For each pH unit, the calculated relative contribution of temperature to fertilization success was C_T_ = 8.29 °C, while the relative contribution of salinity was C_S_ = 10.71 salinity units. These stress values reflect the standardized magnitude of deviation from reference conditions for each variable, scaled by its relative contribution to fertilization success as estimated by slope values from the individual linear regressions. These values were incorporated into the calculation of the total stress (TS) index, which is shown for each fertilization point in relation to fertilization success (Fig. 4). Further details on the total stress values for each observation are provided in Supplementary 3 and in^24^.

## Discussion

Our investigations revealed that temperature and pH, both individually and in combination, significantly influenced fertilization success in *S. droebachiensis*. Specifically, fertilization success generally increased with higher temperatures and was reduced under lower pH conditions. Importantly, the interaction between pH and temperature was significant: fertilization increased more strongly with temperature at high pH, but remained low under low pH conditions even when temperature was elevated. This suggests that warming alone may not fully offset the negative effects of ocean acidification. In contrast, salinity did not have a statistically significant effect on fertilization success, either alone or in combination with temperature. These trends are supported by the fitted interaction model (Supplementary 2) as visualized in Fig. 2.

Fertilization success in sea urchins is influenced by temperature through enhanced sperm motility and by gamete sensitivity to pH. Higher temperatures have been shown to increase sperm swimming speed and metabolic activity, which can improve sperm–egg encounter rates and fertilization success^25^. In contrast, reduced pH can impair intracellular pH regulation in sperm, disrupt the acrosome reaction, and reduce motility by affecting dynein ATPase activity, which is essential for flagellar movement^26,27^. Further, dynein ATPase activity has been observed to increase linearly with intracellular alkalinity in *Strongylocentrotus purpuratus*, clearly linking internal pH (pH_i_) control and motility^28^. More recent evidence from ”kina” *Evechinus chloroticus* demonstrates that ocean acidification causes a failure of sperm to maintain pH_i_, leading to declines in the proportion of motile sperm and swimming speed, which likely reduce fertilization success under near-future acidification scenarios^29^. These physiological impairments under low pH conditions can lower the proportion of motile sperm and slow swimming velocity, ultimately decreasing fertilization success. Such mechanisms are consistent with our observed results, where warming alone could not fully compensate for the negative effects of low pH on fertilization outcomes.

### Simplified performance curves and combined stressors

Performance curves for temperature, pH and salinity are rarely linear and are rather unimodal. However, these curves have a linear part within their tolerance range. We assumed simplified linear performance curves for fertilization success within the tested range of pH, salinity and temperature used in our experiments (Fig. 3). The significant interaction observed between pH and temperature suggests that their combined impact, while generally following the direction of their individual effect, is not mathematically additive consistent from non-linear effects expected from a unimodal performance curve and/or stressors interactions. These findings underscore the importance of experiments covering a wide range of variability to capture the full performance curve.

### Relative contribution of individual drivers

Our results are in line with previous studies that investigated fertilization success in sea urchins. Our regression analysis showed that temperature has a more substantial impact on fertilization success compared to pH. This interpretation is supported by the fitted interaction model (Supplementary 2) and the visualized patterns in Fig. 2, which show differential temperature effects across pH levels.

The main bulk of research on this topic points towards a general consensus in that fertilization in sea urchins is resilient to low pH in the range expected for OA (reviewed in^8^), although some studies have shown contrasting results, even though these seem to be due to differences in methodology. For example, in the tropical shortspined sea urchin (*Heliocidaris erythrogramma*), exposure of gametes to acidification (− 0.4 pH units) negatively affected fertilization success (observed reductions of 24%^30^). Similarly, acidification (800 and 1800 ppm CO_2_) was found to decrease fertilization success at lower sperm concentrations in the temperate red sea urchin (*Strongylocentrotus franciscanus*)^31^. In the sea urchins *Hemicentrotus pulcherrimus* and *Echinometra mathaei*, fertilization rate decreased with increasing *p*CO_2_ concentrations, although the impact differed between individual females^32^.

To standardize the relative effect size of each environmental driver, we extracted slope coefficients from simple linear regressions fitted separately for temperature, pH, and salinity under reference conditions (Supplementary 3). The regressions indicated that fertilization success increased by approximately 2.8% per 1 °C increase in temperature and by 2.3% per 0.1-unit decrease in pH. Expressed differently, a 10% change in fertilization success would correspond to roughly a 3.5 °C increase in temperature or a 0.4-unit decrease in pH. These values provide a more comparable basis for interpreting the relative strength of each driver. Nevertheless, they should not be taken as predictive relationships, since changes of this magnitude may not occur with equal ecological probability. Instead, they offer an approximate measure of relative contributions within the limits of our linear models.

Our results indicate that the coefficient for temperature is higher, indicating its greater influence on reproductive outcomes. This finding is consistent with projections suggesting that rising temperatures will have a more pronounced effects on sea urchin fertilization success than changes in pH alone^11,33^. Previous studies across a range of sea urchin species and thermal environments support the critical role of temperature in determining fertilization success. For example, warming by 2–4 °C enhanced fertilization success in the Antarctic sea urchin *Sterechinus neumayeri*, particularly at low sperm concentrations^34^. Similarly, fertilization in tropical species such as *Echinometra mathaei* and *Toxopneustes roseus* showed strong sensitivity to warming, with success declining sharply above 30°C^35^. In *Lytechinus variegatus*, fertilization was negatively affected by a 3 °C temperature increase, particularly when combined with lowered pH^36^. Conversely, *Heliocidaris erythrogramma*, a temperate species, exhibited resilience to warming up to 6 °C, although developmental impacts became evident at higher thresholds^9^. These results align with our findings that temperature had the strongest effect on fertilization in *S. droebachiensis* and suggest that sensitivity to warming may reflect species-specific thermal tolerances and local adaptation. The contrasting fertilization responses observed across sea urchin species likely reflect differences in physiological adaptation to their native thermal and chemical environments. Polar species such as *S. droebachiensis* and *Sterechinus neumayeri* are adapted to cold, stable conditions and may be more susceptible to rapid warming. In contrast, tropical and temperate species like *Heliocidaris erythrogramma* often tolerate higher temperatures but can be more sensitive to reduced pH, potentially due to species-specific differences in gamete structure and function. For instance, ocean acidification has been shown to impair sperm motility and reduce fertilization success in several echinoid species^34^, while also disrupting intracellular pH regulation and acrosomal function^26^. Conversely, some species show fertilization success to be more strongly influenced by temperature than by pH^9^. These divergent patterns emphasize the role of environmental history, local adaptation, and physiological plasticity in shaping species-specific vulnerabilities to climate change. Sensitivity analysis further supports this, showing that temperature’s coefficient indicates greater sensitivity compared to pH. This underscores the importance of focusing on temperature management in future conservation and mitigation strategies.

### Combined stressor effects

When comparing our results with other studies investigating the effects of multiple stressors (mostly pH, temperature and salinity, although some studies tested contaminants too) on the fertilization success in sea urchins, we observe contrasting results. Similarly to our observations, fertilization in the tropical sea urchin *Pseudoboletia indiana*, significantly decreased with acidification (− 0.3 to 0.5 pH units) while increasing with warming^33^. Other studies have shown no effect of pH or temperature on fertilization success. For example, fertilization success in the tropical sea urchin *H. erythrogramma* was highly dependent on sperm density, and not on temperature (2–4 °C above ambient), pH (0.4–0.6 pH units below ambient), *p*CO_2_ (367–1892 ppm) or their interactions^10^. Similarly, no significant effect of warming and acidification was found on the percentage of fertilization in a suite of tropical echinoids (*H. erythrogramma*, *Heliocidaris tuberculata*, *Tripneustes gratilla* and *Centrostephanus rodgersii*) when exposed to all combinations of three temperatures and three pHs^10^. On the other hand, fertilization in the subtropical sea urchin *Heliocidaris crassispina* was affected by warming (28 °C to 43 °C) and freshening^37^. In another species of subtropical rock-boring sea urchin, *Echinometra lucunter*, fertilization rates were only negatively affected by temperature increase (2 °C), and pH decrease or presence of lead contamination (alone and in combination) did not seem to affect them^38^. In the same species, pH decrease alone negatively affected the fertilization success, even at optimal temperatures^39^. These apparent differences between studies may likely be a consequence of local adaptation to the present range of pH variability^40,41^. In many of the cited studies, ocean acidification scenarios were selected based on IPCC scenarios for open ocean, neglecting the high range of pH variability in coastal areas. As a consequence, some of the tested pH levels fell within the present range, and thus did not represent a true stress condition nor a realistic ocean acidification scenario per se^42^.

The combined effects of temperature, pH, and salinity on fertilization success were explored using a conceptual framework based on standardized slope coefficients from simple linear regressions (Supplementary 3). These ran under reference conditions for each driver, allowed us to compare relative contributions and visualize how individual stressors scale in relation to one another. The results suggest that temperature had the strongest influence on fertilization, followed by pH, while salinity played a minor role. This approach oversimplifies the modelling of the performance curve for these three drivers and only considers its linear part. The observed statistical interactions between temperature and pH support non-linear effects through unimodal performance curves and/or potential stressors interactions. New experiments expanding the pH and temperature range as well as additional information on the drivers’ mode of actions would be needed to fully resolve the combined effect.

### Lessons-learned applied to Arctic regions

It is important to note that this study focuses on a single population of *S. droebachiensis* collected from Kongsfjorden, Svalbard, which may not fully represent the species’ diversity. The green sea urchin has a broad distribution across the North Atlantic and Arctic, including populations in Norway, Greenland, and North America, which experience different environmental conditions. It is therefore possible that other populations may exhibit different sensitivities to temperature and pH based on local adaptation. Additionally, the fertilization assays were conducted using gametes from two females and four males, which limits the genetic diversity represented in the experimental crosses. Fieldwork in polar environments poses substantial logistical and ethical challenges, and the opportunity to collect and experimentally test high numbers of individuals and multiple populations is often constrained by weather, accessibility, and seasonal availability. Despite these limitations, our findings provide critical data points for Arctic populations and can serve as a baseline for future comparative studies aimed at understanding intraspecific variability across broader spatial and environmental gradients.

Research in polar sea urchins is rather limited, and the few available studies show contrasting results. Fertilization in the Antarctic sea urchin *Sterechinus neumayeri* was resilient to acidification (pH ~ 7.7 and 7.5) at ambient temperature (0 °C), but not under elevated temperatures (1.5 °C and 3 °C), where it was negatively impacted by both drivers independently as well as by their interactions^43^. However, when tested at a range of sperm concentrations, warming (1 °C, 3 °C and 5 °C; 2–4 °C above ambient) enhanced fertilization in the same sea urchin *S. neumayeri* at the lowest sperm concentrations, whereas decreased pH (pH 8.0, pH 7.8 and pH 7.6; 0.2–0.4 pH units below ambient) did not have any effect^34^. Another study of the same species found reduced fertilization success under environmental-relevant OA scenarios although the responses observed indicated a high degree of individual variability^44^. Even less information is available on Arctic species of sea urchins, and the only available study focused on the effects of acidification alone. Fertilization in *S. droebachiensis* collected from Kongsfjorden (Svalbard) was impaired by acidification, decreasing significantly at extreme levels of acidification (~ 2000 µatm *p*CO_2_) but not at the other levels of acidification tested^45^, results that contrast with our observations. Although some Antarctic sea urchins have shown resilience to OA when tested in isolation, their fertilization success may still be negatively affected under combined stressors such as warming and acidification, suggesting limited overall resilience under realistic environmental conditions.

## Conclusions

Our investigation provides insights into the complexity of the relation between environmental stressors and the responses in marine organisms. While our observations align with previous research, it also contrasts with other studies. The variability in responses underscores the difficulties in providing definitive conclusions about the impacts of multiple stressors. This variability is not only present among populations but also among individuals, reflecting the complex nature of multiple stressors and their effects on marine organisms. Our results corroborate the notion that multiple stressor investigations are inherently complicated, and the relationships among stressors are often difficult to discern.

Despite these complexities, our study emphasizes the critical need for continued research on organismal and ecosystem responses to multiple stressors, particularly in polar ecosystems. Given that environmental changes are occurring at an accelerated pace in polar regions compared to other parts of the world, understanding these mechanisms is crucial. This research is timely and necessary to develop effective management and conservation strategies for polar marine ecosystems, ensuring their resilience in the face of rapid environmental changes.

## Supplementary Information

Below is the link to the electronic supplementary material.

Supplementary Material 1

## Data Availability

**Supporting data for this study is available at 10.21334/NPOLAR.2024.9A45CB17 (fertilization data; 21) and 10.21334/NPOLAR.2025.9BF189E2 (calculations of the stress index and individual contributions; 24).**.
