# Supplementary material for: Combined effects of ocean acidification, warming, and salinity on the fertilization success in an Arctic population of sea urchins
Source: Sci Rep. 2025 Dec 18;15:44090. doi: 10.1038/s41598-025-27725-z (PMC12715215; doi:10.1038/s41598-025-27725-z)
Supplement: Supplementary file 1 — Supplementary Material 1 [file 41598_2025_27725_MOESM1_ESM.pdf]

Combined effects of ocean acidification, warming, and salinity on the fertilization success in an Arctic population of the green sea urchin (*Strongylocentrotus droebachiensis*)

Nadjejda Espinel-Velasco[1,2*], Ane Cecilie Kvernvik[1], Haakon Hop[1], Sam Dupont[3,4]

[1] Norwegian Polar Institute, Fram Centre, 9296 Tromsø, Norway

[2] Department of Marine Science, University of Gothenburg, Tjärno Marine Laboratory, Sweden

[3] Department of Biological and Environmental Sciences, University of Gothenburg, Kristineberg, Sweden

[4] Marine and Freshwater Research Institute, Fornubudir 4, 220 Hafnarfjörður, Iceland

*Corresponding author: nadjejda.espinel@gu.se

SUPPLEMENTARY MATERIAL: Fertilization success and multiple stressors

Supplementary 1 – pilot fertilization assay

For the pilot fertilization assay, 5 concentrations of sperm were used in 4 replicates. The assays were done in 24 multiwell plates, where each row of 4 represented a concentration. Each well was loaded with approximately 200 eggs.

The first sperm concentration (conc. 1) was prepared by ading 20 µl of sperm to 80 µl of seawater. Each of the 4 wells (replicates) for concentration 1 was loaded with 20 µl of this concentration. Concentration 2 was prepared by diluting 10 times the first concentration 1 (by mixing 10 µl of conc. 1 with 90 µl of FSW). The following concentrations were prepared by subsequently diluting 10 times the previous concentration, until arriving at concentration 5. Subsequently, 20 µl from each concentration was added into the respective wells. After 15 min, a drop of 4% PFA solution was added to each well to stop the process and fix the eggs. Subsequently, the eggs in each well were scored under a microscope to determine the fertilization success (see table below). Eggs that are fertilized present a perivitelline membrane that is easily recognizable under the microscope. Concentration 2 was deemed the most appropriate for the fertilization assays, given that it led to about 50% of fertilization success (45.65%, see table and plot below).

SUPPLEMENTARY MATERIAL: Fertilization success and multiple stressors

	concentration 1			concentration 2			concentration 3			concentration 4			concentration 5		
Replicates	fertilized	unfertilized	% fertilization	fertilized	unfertilized	% fertilization	fertilized	unfertilized	% fertilization	fertilized	unfertilized	% fertilization	fertilized	unfertilized	% fertilization
r1	148	2	98.67	46	86	34.85	19	122	13.48	2	150	1.32	0	71	0.00
r2	111	10	91.74	112	55	67.07	41	112	26.80	6	161	3.59	0	172	0.00
r3	144	6	96.00	60	88	40.54	32	139	18.71	5	171	2.84	0	155	0.00
r4	122	24	83.56	49	73	40.16	29	124	18.95	7	165	4.07	0	201	0.00
mean	131.25	10.50	**92.49**	66.75	75.50	**45.65**	30.25	124.25	**19.49**	5.00	161.75	**2.95**	0.00	149.75	**0.00**
SD	17.69	9.57	**6.60**	30.76	15.20	**14.51**	9.07	11.15	**5.49**	2.16	8.85	**1.20**	0.00	55.83	**0.00**
SEM	8.84	4.79	**3.30**	15.38	7.60	**7.25**	4.53	5.57	**2.75**	1.08	4.42	**0.60**	0.00	27.91	**0.00**

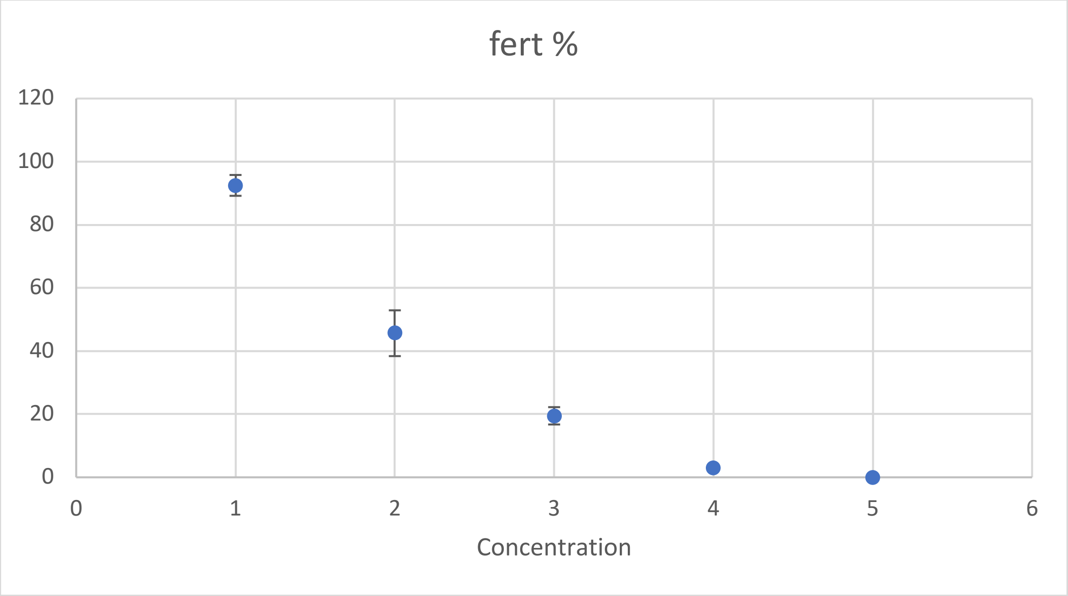

Supplementary 2 – Model selection and validation plots

One model to test for results – all variables but no interaction pH×temp, since there is no crossed data for them

Building the model

lm(perc.fert ~ temperature:pH + salinity:temperature + salinity+temperature+pH, data = fert)

```
Call:
lm(formula = perc.fert ~ temperature:pH + temperature:salinity +
    pH + salinity + temperature, data = fert)

Residuals:
    Min      1Q  Median      3Q     Max
-47.056  -6.133   2.722   7.683  25.027

Coefficients:
                     Estimate Std. Error t value Pr(>|t|)
(Intercept)         -84.47822   51.18542  -1.650 0.101128
pH                   18.07229    4.41678   4.092 7.25e-05 ***
salinity              0.03929    0.75104   0.052 0.958359
temperature         -26.41299   13.21896  -1.998 0.047669 *
temperature:pH        4.08506    1.14066   3.581 0.000473 ***
temperature:salinity -0.12633    0.19396  -0.651 0.515918
---
Signif. codes:  0 ‘***’ 0.001 ‘**’ 0.01 ‘*’ 0.05 ‘.’ 0.1 ‘ ’ 1

Residual standard error: 11.53 on 138 degrees of freedom
Multiple R-squared:  0.5627,    Adjusted R-squared:  0.5468
F-statistic: 35.51 on 5 and 138 DF,  p-value: < 2.2e-16
```

We can see that *p*-value of the F-statistic is < 2.2e-16, which is highly significant. This implies that, at least, one of the predictor variables is significantly related to the outcome variable.

To determine which predictor variables are significant, we can examine the coefficients table, which shows the estimate of regression beta coefficients and the associated *t*-statistic *p*-values:

Interpretation

```
> summary(test_lm)$coefficient
                        Estimate Std. Error     t value     Pr(>|t|)
(Intercept)          -84.47821650 51.1854200 -1.65043515 1.011281e-01
pH                    18.07228755  4.4167773  4.09173622 7.246271e-05
salinity               0.03928552  0.7510401  0.05230815 9.583588e-01
temperature          -26.41299192 13.2189565 -1.99811475 4.766950e-02
temperature:pH         4.08505796  1.1406605  3.58130923 4.727319e-04
temperature:salinity  -0.12633225  0.1939608 -0.65132864 5.159175e-01
```

Changes in pH, temperature and the interactions of temperature: pH are significantly associated with changes in F! success, but not salinity or the interaction of

temp×salinity. Since these both were not significant, they were removed from the model.

Clean model

```
Call:
lm(formula = perc.fert ~ temperature:pH + pH + temperature, data = fert)

Residuals:
    Min      1Q  Median      3Q     Max 
-46.115  -7.393   2.962   7.714  25.115 

Coefficients:
               Estimate Std. Error t value Pr(>|t|)    
(Intercept)    -82.3187    30.1320  -2.732  0.00711 ** 
pH              17.9597     3.8403   4.677 6.79e-06 ***
temperature    -33.3574     7.7818  -4.287 3.36e-05 ***
temperature:pH   4.4472     0.9918   4.484 1.51e-05 ***
---
Signif. codes:  0 ‘***’ 0.001 ‘**’ 0.01 ‘*’ 0.05 ‘.’ 0.1 ‘ ’ 1

Residual standard error: 11.48 on 140 degrees of freedom
Multiple R-squared:   0.56,	Adjusted R-squared:  0.5506 
F-statistic:  59.4 on 3 and 140 DF,  p-value: < 2.2e-16
```

```
> summary(test_lm)$coefficient
                 Estimate Std. Error   t value     Pr(>|t|)
(Intercept)    -82.318720 30.1319842 -2.731938 7.107757e-03
pH              17.959660  3.8403004  4.676629 6.794178e-06
temperature    -33.357385  7.7817743 -4.286604 3.356740e-05
temperature:pH   4.447239  0.9917817  4.484091 1.512419e-05
```

The model equation can then be written as:

F! = - 82.32 - 33.36*Temperature + 17.96*pH + 4.45 (Temperature*pH)

Confidence interval of the model coefficient:

```
> confint(test_lm)
                     2.5 %     97.5 %
(Intercept)    -141.891271 -22.746168
pH               10.367180  25.552140
temperature     -48.742371 -17.972399
temperature:pH    2.486433   6.408045
```

Model accuracy assessment

R-squared

SUPPLEMENTARY MATERIAL: Fertilization success and multiple stressors

The new clean model has a (slight) better adjusted R^2 (this takes into account the number of predictor variables) of 0.55, meaning 55% of the variance in the measure of F! can be predicted by pH and temp and their interactions.

Residual Standard Error (RSE) or sigma

```
> sigma(test_lm)/mean(fert$perc.fert)
[1] 0.1830086
```

Our RSE of 11.48 corresponds with 18% error rate.

This model shows that temperature and pH have a strong effect on the fertilization success, but not salinity. Also, strong interaction between temperature and pH, but not between temperature and salinity.

Check the model

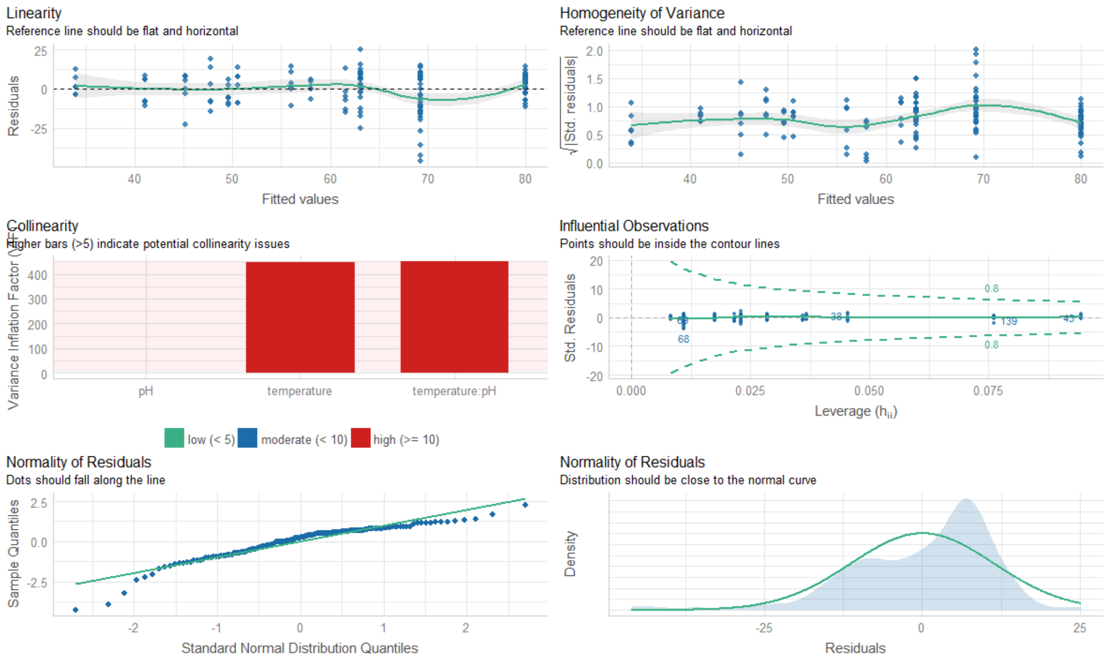

The chosen model shows strong potential multicollinearity of the structural type. The interaction term of temperature×pH is included.

SUPPLEMENTARY MATERIAL: Fertilization success and multiple stressors

Clearly, there is a correlation between the interaction term and both of the main effect terms and the Variance Inflation Factors (VIFs) reflect these relationships.

Supplementary 3 – calculations of the total stress for each observation

We used three models to build the index with the relative contribution of the drivers:

a) Model 1 = Temperature at pH 8.1 and salinity 34 ->

```
Call:
lm(formula = perc.fert ~ temperature, data = fert.ambtemp)

Residuals:
     Min       1Q   Median       3Q      Max
-21.7297  -7.8007  -0.4439   7.5529  28.6903

Coefficients:
            Estimate Std. Error t value Pr(>|t|)
(Intercept)  59.5797     2.6090  22.836  < 2e-16 ***
temperature   2.8091     0.6738   4.169 0.000199 ***
---
Signif. codes:  0 ‘***’ 0.001 ‘**’ 0.01 ‘*’ 0.05 ‘.’ 0.1 ‘ ’ 1

Residual standard error: 10.52 on 34 degrees of freedom
Multiple R-squared:  0.3383,	Adjusted R-squared:  0.3188
F-statistic: 17.38 on 1 and 34 DF,  p-value: 0.0001989
```

Model 1 equation -> F! = 59.58 + 2.81*Temperature

R2 = 0.34   Adjusted R^2 = 0.32 -> 32% of variance is explained by temperature

Confidence interval of the model coefficient

```
> confint(lm_ambtemp)
                2.5 %    97.5 %
(Intercept) 54.277593 64.881822
temperature  1.439809  4.178418
```

Residual Standard Error (RSE) or sigma (the RSE of 10.52 corresponds with 16% error rate)

```
> sigma(lm_ambtemp)/mean(fert$perc.fert)
[1] 0.1677432
```

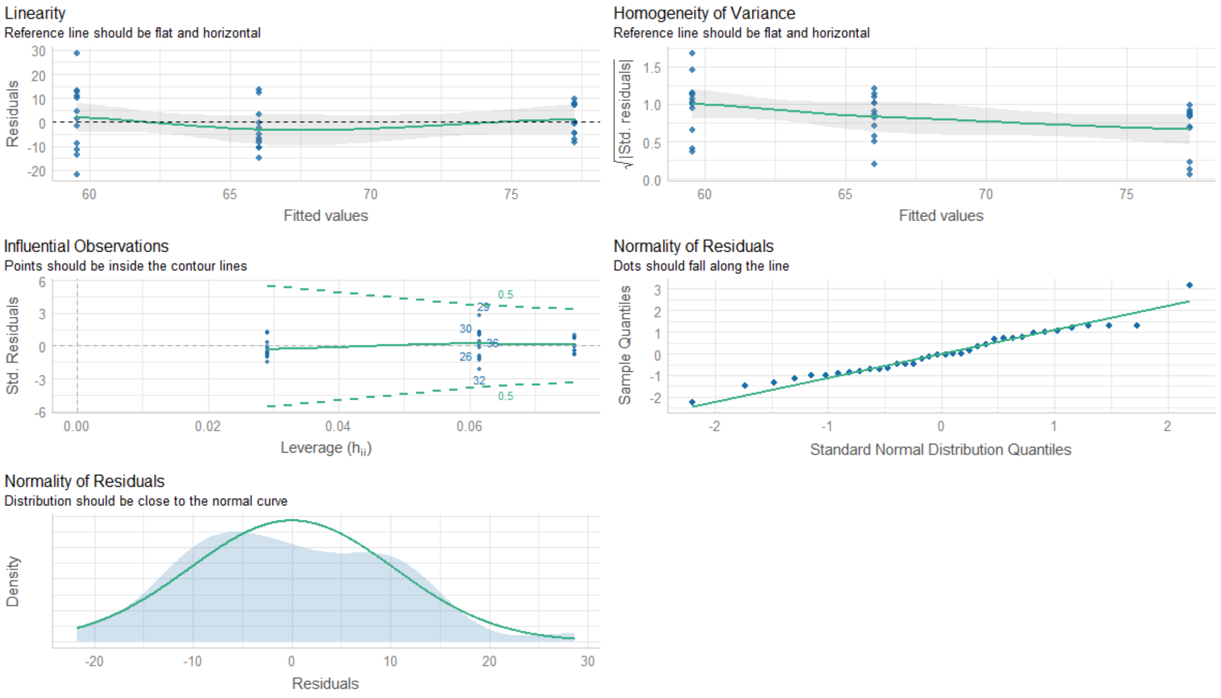

b) Model 2 = Salinity at pH 8.1 and temp 2.2

i. Select data

ii. Model ➔ lm (perc.fert ~ salinity, data= fert.ambsal)

iii. Obtain R^2 and equation

```
Call:
lm(formula = perc.fert ~ salinity, data = fert.ambsal)

Residuals:
    Min      1Q  Median      3Q     Max
-31.004  -9.377  -2.655  12.328  28.646

Coefficients:
            Estimate Std. Error t value Pr(>|t|)
(Intercept)   -6.728     38.465  -0.175    0.862
salinity       2.175      1.214   1.792    0.084 .
---
Signif. codes:  0 ‘***’ 0.001 ‘**’ 0.01 ‘*’ 0.05 ‘.’ 0.1 ‘ ’ 1

Residual standard error: 15.51 on 28 degrees of freedom
Multiple R-squared:  0.1029,	Adjusted R-squared:  0.07082
F-statistic:  3.21 on 1 and 28 DF,  p-value: 0.08399
```

Model 2 equation -> F! = -6.73 + 2.18*Salinity

R2 = 0.10 Adjusted R2=0.07 -> 7% of variance is explained by temperature

Confidence interval of the model coefficient

```
> confint(lm_ambsal)
                  2.5 %    97.5 %
(Intercept) -85.5189540 72.062974
salinity     -0.3115472  4.661694
```

Residual Standard Error (RSE) or sigma
(the RSE of 15.51 corresponds with 24% error rate)

```
> sigma(lm_ambsal)/mean(fert$perc.fert)
[1] 0.2472012
```

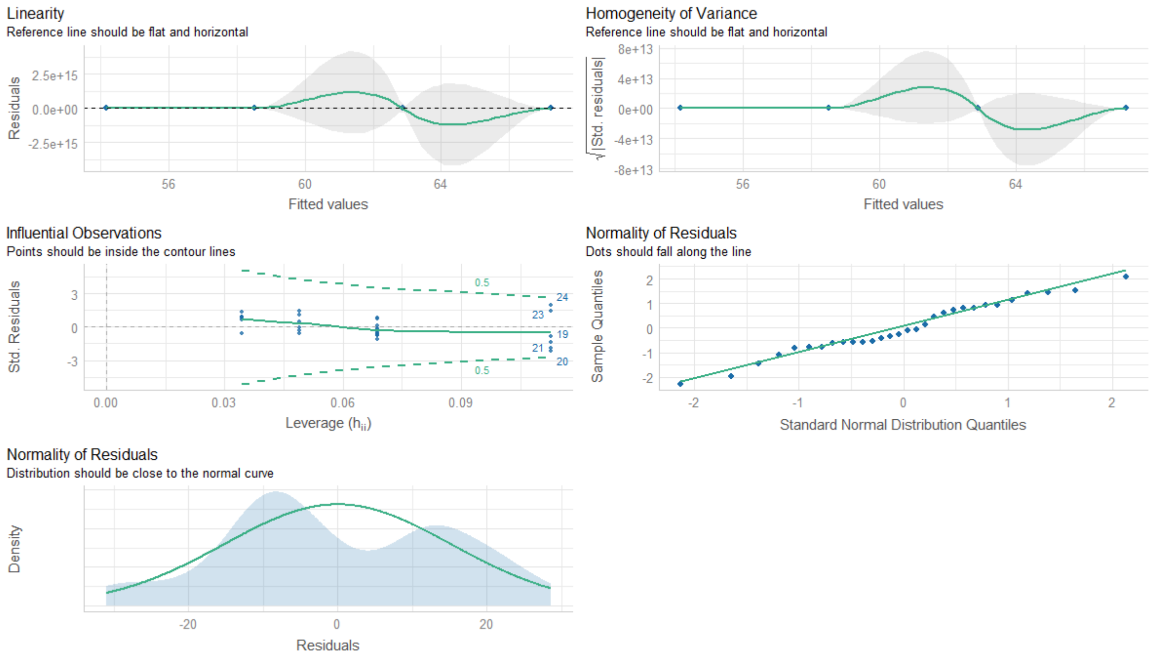

c) Model 3 = pH at salinity 34 and temp 2.2

```
Call:
lm(formula = perc.fert ~ pH, data = fert.ambpH)

Residuals:
    Min       1Q  Median      3Q     Max 
-12.774  -7.004  -1.689   6.253  15.676 

Coefficients:
            Estimate Std. Error t value Pr(>|t|)    
(Intercept) -124.837     27.936  -4.469 0.000118 ***
pH            23.300      3.633   6.414 6.05e-07 ***
---
Signif. codes:  0 ‘***’ 0.001 ‘**’ 0.01 ‘*’ 0.05 ‘.’ 0.1 ‘ ’ 1

Residual standard error: 7.798 on 28 degrees of freedom
Multiple R-squared:  0.595,	Adjusted R-squared:  0.5806 
F-statistic: 41.14 on 1 and 28 DF,  p-value: 6.053e-07
```

Model 3 equation -> F! = -124.84 + 23.30*pH

R2 = 0.59   Adjusted R^2=0.58 -> 58% of variance is explained by temperature

Confidence interval of the model coefficient

```
> confint(lm_ambpH)
                 2.5 %    97.5 %
(Intercept) -182.06049 -67.61284
pH            15.85875  30.74142
```

Residual Standard Error (RSE) or sigma
(the RSE of 15.51 corresponds with 24% error rate)

```
> sigma(lm_ambpH)/mean(fert$perc.fert)
[1] 0.1243049
```

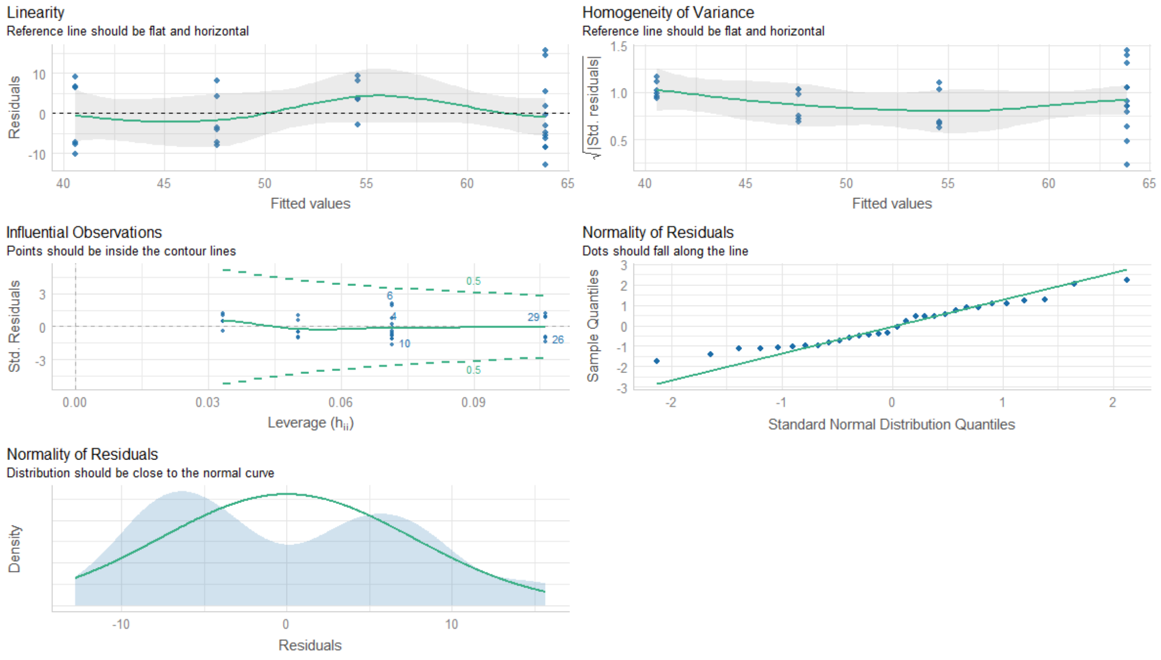

We then calculated the relative contribution of each driver:

Model 1 equation: Temperature -> F! = 59.58 + 2.81x

Model 2 equation: Salinity -> F! = -6.73 + 2.18x

Model 3 equation: pH -> F! = -124.84 + 23.30x

Contribution of temperature:

23.301/2.8094 = **8.29 °C**

Contribution of Salinity:

23.301/2.1751 = **10.71 psu**

Finally, we built the index with the relative contributions of them all at 2.2 °C

The table below shows the calculated values of total stress for each observation.

plate	rep	S	pH	temp	fert	unfert	% fert	stress unit pH	stress unit temp 6.3	stress unit temp 2.2	stress unit salinity 6.3	stress unit salinity (2.2°C)	Total stress	% fert	Total stress 3 (temp 6.3)	Total stress 3 (temp 2.2)	% fert
20	1	28	8.10	0.0	120	46	72.29	0.00	-0.42	-0.2774	0.2063	2.4183	-0.4200	72.29	-0.2137	2.1409	72.29
20	2	28	8.10	0.0	107	38	73.79	0.00	-0.42	-0.2774	0.2063	2.4183	-0.4200	73.79	-0.2137	2.1409	73.79
20	3	28	8.10	0.0	98	41	70.50	0.00	-0.42	-0.2774	0.2063	2.4183	-0.4200	70.50	-0.2137	2.1409	70.50
20	4	28	8.10	0.0	95	34	73.64	0.00	-0.42	-0.2774	0.2063	2.4183	-0.4200	73.64	-0.2137	2.1409	73.64
20	5	28	8.10	0.0	94	40	70.15	0.00	-0.42	-0.2774	0.2063	2.4183	-0.4200	70.15	-0.2137	2.1409	70.15
20	6	28	8.10	0.0	93	59	61.18	0.00	-0.42	-0.2774	0.2063	2.4183	-0.4200	61.18	-0.2137	2.1409	61.18
12	1	28	8.10	2.2	52	103	33.55	0.00	-0.27	0.0000	0.2063	2.4183	-0.2667	33.55	-0.0604	2.4183	33.55
12	2	28	8.10	2.2	38	126	23.17	0.00	-0.27	0.0000	0.2063	2.4183	-0.2667	23.17	-0.0604	2.4183	23.17
12	3	28	8.10	2.2	44	120	26.83	0.00	-0.27	0.0000	0.2063	2.4183	-0.2667	26.83	-0.0604	2.4183	26.83
12	4	28	8.10	2.2	85	117	42.08	0.00	-0.27	0.0000	0.2063	2.4183	-0.2667	42.08	-0.0604	2.4183	42.08
12	5	28	8.10	2.2	144	46	75.79	0.00	-0.27	0.0000	0.2063	2.4183	-0.2667	75.79	-0.0604	2.4183	75.79
12	6	28	8.10	2.2	135	28	82.82	0.00	-0.27	0.0000	0.2063	2.4183	-0.2667	82.82	-0.0604	2.4183	82.82
4	1	28	8.10	6.3	135	31	81.33	0.00	0.0000	0.4825	0.2063	2.4183	0.0000	81.33	0.2063	2.9008	81.33
4	2	28	8.10	6.3	154	19	89.02	0.00	0.0000	0.4825	0.2063	2.4183	0.0000	89.02	0.2063	2.9008	89.02
4	3	28	8.10	6.3	166	10	94.32	0.00	0.0000	0.4825	0.2063	2.4183	0.0000	94.32	0.2063	2.9008	94.32
4	4	28	8.10	6.3	171	33	83.82	0.00	0.0000	0.4825	0.2063	2.4183	0.0000	83.82	0.2063	2.9008	83.82
4	5	28	8.10	6.3	155	38	80.31	0.00	0.0000	0.4825	0.2063	2.4183	0.0000	80.31	0.2063	2.9008	80.31
4	6	28	8.10	6.3	107	18	85.60	0.00	0.0000	0.4825	0.2063	2.4183	0.0000	85.60	0.2063	2.9008	85.60
19	1	30	8.10	0.0	133	42	76.00	0.00	-0.4200	-0.2774	0.1375	2.4183	-0.4200	76.00	-0.2825	2.1409	76.00
19	2	30	8.10	0.0	118	42	73.75	0.00	-0.4200	-0.2774	0.1375	2.4183	-0.4200	73.75	-0.2825	2.1409	73.75
19	3	30	8.10	0.0	146	39	78.92	0.00	-0.4200	-0.2774	0.1375	2.4183	-0.4200	78.92	-0.2825	2.1409	78.92
19	4	30	8.10	0.0	92	48	65.71	0.00	-0.4200	-0.2774	0.1375	2.4183	-0.4200	65.71	-0.2825	2.1409	65.71

SUPPLEMENTARY MATERIAL: Fertilization success and multiple stressors

plate	rep	S	pH	temp	fert	unfert	% fert	stress unit pH	stress unit temp 6.3	stress unit temp 2.2	stress unit salinity 6.3	stress unit salinity (2.2°C)	Total stress	% fert	Total stress 3 (temp 6.3)	Total stress 3 (temp 2.2)	% fert
19	5	30	8.10	0.0	112	41	73.20	0.00	-0.4200	-0.2774	0.1375	2.4183	-0.4200	73.20	-0.2825	2.1409	73.20
19	6	30	8.10	0.0	113	48	70.19	0.00	-0.4200	-0.2774	0.1375	2.4183	-0.4200	70.19	-0.2825	2.1409	70.19
11	1	30	8.10	2.2	78	79	49.68	0.00	-0.2667	0.0000	0.1375	2.4183	-0.2667	49.68	-0.1292	2.4183	49.68
11	2	30	8.10	2.2	89	78	53.29	0.00	-0.2667	0.0000	0.1375	2.4183	-0.2667	53.29	-0.1292	2.4183	53.29
11	3	30	8.10	2.2	92	68	57.50	0.00	-0.2667	0.0000	0.1375	2.4183	-0.2667	57.50	-0.1292	2.4183	57.50
11	4	30	8.10	2.2	132	43	75.43	0.00	-0.2667	0.0000	0.1375	2.4183	-0.2667	75.43	-0.1292	2.4183	75.43
11	5	30	8.10	2.2	105	56	65.22	0.00	-0.2667	0.0000	0.1375	2.4183	-0.2667	65.22	-0.1292	2.4183	65.22
11	6	30	8.10	2.2	146	37	79.78	0.00	-0.2667	0.0000	0.1375	2.4183	-0.2667	79.78	-0.1292	2.4183	79.78
3	1	30	8.10	6.3	164	14	92.13	0.00	0.0000	0.4825	0.1375	2.4183	0.0000	92.13	0.1375	2.9008	92.13
3	2	30	8.10	6.3	160	20	88.89	0.00	0.0000	0.4825	0.1375	2.4183	0.0000	88.89	0.1375	2.9008	88.89
3	3	30	8.10	6.3	139	23	85.80	0.00	0.0000	0.4825	0.1375	2.4183	0.0000	85.80	0.1375	2.9008	85.80
3	4	30	8.10	6.3	137	23	85.63	0.00	0.0000	0.4825	0.1375	2.4183	0.0000	85.63	0.1375	2.9008	85.63
3	5	30	8.10	6.3	155	17	90.12	0.00	0.0000	0.4825	0.1375	2.4183	0.0000	90.12	0.1375	2.9008	90.12
3	6	30	8.10	6.3	122	19	86.52	0.00	0.0000	0.4825	0.1375	2.4183	0.0000	86.52	0.1375	2.9008	86.52
18	1	32	8.10	0.0	134	51	72.43	0.00	-0.4200	-0.2774	0.0688	2.4183	-0.4200	72.43	-0.3512	2.1409	72.43
18	2	32	8.10	0.0	120	61	66.30	0.00	-0.4200	-0.2774	0.0688	2.4183	-0.4200	66.30	-0.3512	2.1409	66.30
18	3	32	8.10	0.0	121	45	72.89	0.00	-0.4200	-0.2774	0.0688	2.4183	-0.4200	72.89	-0.3512	2.1409	72.89
18	4	32	8.10	0.0	103	39	72.54	0.00	-0.4200	-0.2774	0.0688	2.4183	-0.4200	72.54	-0.3512	2.1409	72.54
18	5	32	8.10	0.0	32	14	69.57	0.00	-0.4200	-0.2774	0.0688	2.4183	-0.4200	69.57	-0.3512	2.1409	69.57
18	6	32	8.10	0.0	128	57	69.19	0.00	-0.4200	-0.2774	0.0688	2.4183	-0.4200	69.19	-0.3512	2.1409	69.19
10	1	32	8.10	2.2	77	65	54.23	0.00	-0.2667	0.0000	0.0688	2.4183	-0.2667	54.23	-0.1979	2.4183	54.23
10	2	32	8.10	2.2	130	39	76.92	0.00	-0.2667	0.0000	0.0688	2.4183	-0.2667	76.92	-0.1979	2.4183	76.92

SUPPLEMENTARY MATERIAL: Fertilization success and multiple stressors

plate	rep	S	pH	temp	fert	unfert	% fert	stress unit pH	stress unit temp 6.3	stress unit temp 2.2	stress unit salinity 6.3	stress unit salinity (2.2°C)	Total stress	% fert	Total stress 3 (temp 6.3)	Total stress 3 (temp 2.2)	% fert
10	3	32	8.10	2.2	80	15	84.21	0.00	-0.2667	0.0000	0.0688	2.4183	-0.2667	84.21	-0.1979	2.4183	84.21
10	4	32	8.10	2.2	127	42	75.15	0.00	-0.2667	0.0000	0.0688	2.4183	-0.2667	75.15	-0.1979	2.4183	75.15
10	5	32	8.10	2.2	148	44	77.08	0.00	-0.2667	0.0000	0.0688	2.4183	-0.2667	77.08	-0.1979	2.4183	77.08
10	6	32	8.10	2.2	119	45	72.56	0.00	-0.2667	0.0000	0.0688	2.4183	-0.2667	72.56	-0.1979	2.4183	72.56
2	1	32	8.10	6.3	145	36	80.11	0.00	0.0000	0.4825	0.0688	2.4183	0.0000	80.11	0.0688	2.9008	80.11
2	2	32	8.10	6.3	162	28	85.26	0.00	0.0000	0.4825	0.0688	2.4183	0.0000	85.26	0.0688	2.9008	85.26
2	3	32	8.10	6.3	133	27	83.13	0.00	0.0000	0.4825	0.0688	2.4183	0.0000	83.13	0.0688	2.9008	83.13
2	4	32	8.10	6.3	129	18	87.76	0.00	0.0000	0.4825	0.0688	2.4183	0.0000	87.76	0.0688	2.9008	87.76
2	5	32	8.10	6.3	141	22	86.50	0.00	0.0000	0.4825	0.0688	2.4183	0.0000	86.50	0.0688	2.9008	86.50
2	6	32	8.10	6.3	126	17	88.11	0.00	0.0000	0.4825	0.0688	2.4183	0.0000	88.11	0.0688	2.9008	88.11
24	1	34	7.10	0.0	34	117	22.52	-1.00	-0.4200	-0.2774	0.0000	2.5117	-1.4200	22.52	-1.4200	1.2342	22.52
24	2	34	7.10	0.0	87	77	53.05	-1.00	-0.4200	-0.2774	0.0000	2.5117	-1.4200	53.05	-1.4200	1.2342	53.05
24	3	34	7.10	0.0	80	78	50.63	-1.00	-0.4200	-0.2774	0.0000	2.5117	-1.4200	50.63	-1.4200	1.2342	50.63
24	4	34	7.10	0.0	71	87	44.94	-1.00	-0.4200	-0.2774	0.0000	2.5117	-1.4200	44.94	-1.4200	1.2342	44.94
24	5	34	7.10	0.0	56	76	42.42	-1.00	-0.4200	-0.2774	0.0000	2.5117	-1.4200	42.42	-1.4200	1.2342	42.42
24	6	34	7.10	0.0	74	64	53.62	-1.00	-0.4200	-0.2774	0.0000	2.5117	-1.4200	53.62	-1.4200	1.2342	53.62
16	1	34	7.10	2.2	55	110	33.33	-1.00	-0.2667	0.0000	0.0000	2.5117	-1.2667	33.33	-1.2667	1.5117	33.33
16	2	34	7.10	2.2	65	148	30.52	-1.00	-0.2667	0.0000	0.0000	2.5117	-1.2667	30.52	-1.2667	1.5117	30.52
16	3	34	7.10	2.2	70	78	47.30	-1.00	-0.2667	0.0000	0.0000	2.5117	-1.2667	47.30	-1.2667	1.5117	47.30
16	4	34	7.10	2.2	56	114	32.94	-1.00	-0.2667	0.0000	0.0000	2.5117	-1.2667	32.94	-1.2667	1.5117	32.94
16	5	34	7.10	2.2	83	84	49.70	-1.00	-0.2667	0.0000	0.0000	2.5117	-1.2667	49.70	-1.2667	1.5117	49.70
16	6	34	7.10	2.2	73	82	47.10	-1.00	-0.2667	0.0000	0.0000	2.5117	-1.2667	47.10	-1.2667	1.5117	47.10

SUPPLEMENTARY MATERIAL: Fertilization success and multiple stressors

plate	rep	S	pH	temp	fert	unfert	% fert	stress unit pH	stress unit temp 6.3	stress unit temp 2.2	stress unit salinity 6.3	stress unit salinity (2.2°C)	Total stress	% fert	Total stress 3 (temp 6.3)	Total stress 3 (temp 2.2)	% fert
8	1	34	7.10	6.3	78	90	46.43	-1.00	0.0000	0.4825	0.0000	2.5117	-1.0000	46.43	-1.0000	1.9942	46.43
8	2	34	7.10	6.3	63	115	35.39	-1.00	0.0000	0.4825	0.0000	2.5117	-1.0000	35.39	-1.0000	1.9942	35.39
8	3	34	7.10	6.3	55	127	30.22	-1.00	0.0000	0.4825	0.0000	2.5117	-1.0000	30.22	-1.0000	1.9942	30.22
8	4	34	7.10	6.3	58	107	35.15	-1.00	0.0000	0.4825	0.0000	2.5117	-1.0000	35.15	-1.0000	1.9942	35.15
8	5	34	7.10	6.3	50	114	30.49	-1.00	0.0000	0.4825	0.0000	2.5117	-1.0000	30.49	-1.0000	1.9942	30.49
8	6	34	7.10	6.3	62	88	41.33	-1.00	0.0000	0.4825	0.0000	2.5117	-1.0000	41.33	-1.0000	1.9942	41.33
23	1	34	7.40	0.0	76	107	41.53	-0.70	-0.4200	-0.2774	0.0000	2.4837	-1.1200	41.53	-1.1200	1.5062	41.53
23	2	34	7.40	0.0	81	58	58.27	-0.70	-0.4200	-0.2774	0.0000	2.4837	-1.1200	58.27	-1.1200	1.5062	58.27
23	3	34	7.40	0.0	80	71	52.98	-0.70	-0.4200	-0.2774	0.0000	2.4837	-1.1200	52.98	-1.1200	1.5062	52.98
23	4	34	7.40	0.0	122	68	64.21	-0.70	-0.4200	-0.2774	0.0000	2.4837	-1.1200	64.21	-1.1200	1.5062	64.21
23	5	34	7.40	0.0	68	96	41.46	-0.70	-0.4200	-0.2774	0.0000	2.4837	-1.1200	41.46	-1.1200	1.5062	41.46
23	6	34	7.40	0.0	75	54	58.14	-0.70	-0.4200	-0.2774	0.0000	2.4837	-1.1200	58.14	-1.1200	1.5062	58.14
15	1	34	7.40	2.2	62	92	40.26	-0.70	-0.2667	0.0000	0.0000	2.4837	-0.9667	40.26	-0.9667	1.7837	40.26
15	2	34	7.40	2.2	96	89	51.89	-0.70	-0.2667	0.0000	0.0000	2.4837	-0.9667	51.89	-0.9667	1.7837	51.89
15	3	34	7.40	2.2	80	102	43.96	-0.70	-0.2667	0.0000	0.0000	2.4837	-0.9667	43.96	-0.9667	1.7837	43.96
15	4	34	7.40	2.2	72	93	43.64	-0.70	-0.2667	0.0000	0.0000	2.4837	-0.9667	43.64	-0.9667	1.7837	43.64
15	5	34	7.40	2.2	54	43	55.67	-0.70	-0.2667	0.0000	0.0000	2.4837	-0.9667	55.67	-0.9667	1.7837	55.67
15	6	34	7.40	2.2	57	87	39.58	-0.70	-0.2667	0.0000	0.0000	2.4837	-0.9667	39.58	-0.9667	1.7837	39.58
7	1	34	7.40	6.3	105	66	61.40	-0.70	0.0000	0.4825	0.0000	2.4837	-0.7000	61.40	-0.7000	2.2662	61.40
7	2	34	7.40	6.3	120	60	66.67	-0.70	0.0000	0.4825	0.0000	2.4837	-0.7000	66.67	-0.7000	2.2662	66.67
7	3	34	7.40	6.3	94	92	50.54	-0.70	0.0000	0.4825	0.0000	2.4837	-0.7000	50.54	-0.7000	2.2662	50.54

SUPPLEMENTARY MATERIAL: Fertilization success and multiple stressors

plate	rep	S	pH	temp	fert	unfert	% fert	stress unit pH	stress unit temp 6.3	stress unit temp 2.2	stress unit salinity 6.3	stress unit salinity (2.2°C)	Total stress	% fert	Total stress 3 (temp 6.3)	Total stress 3 (temp 2.2)	% fert
7	4	34	7.40	6.3	54	108	33.33	-0.70	0.0000	0.4825	0.0000	2.4837	-0.7000	33.33	-0.7000	2.2662	33.33
7	5	34	7.40	6.3	64	98	39.51	-0.70	0.0000	0.4825	0.0000	2.4837	-0.7000	39.51	-0.7000	2.2662	39.51
7	6	34	7.40	6.3	65	97	40.12	-0.70	0.0000	0.4825	0.0000	2.4837	-0.7000	40.12	-0.7000	2.2662	40.12
22	1	34	7.70	0.0	101	77	56.74	-0.40	-0.4200	-0.2774	0.0000	2.4556	-0.8200	56.74	-0.8200	1.7782	56.74
22	2	34	7.70	0.0	97	77	55.75	-0.40	-0.4200	-0.2774	0.0000	2.4556	-0.8200	55.75	-0.8200	1.7782	55.75
22	3	34	7.70	0.0	92	62	59.74	-0.40	-0.4200	-0.2774	0.0000	2.4556	-0.8200	59.74	-0.8200	1.7782	59.74
22	4	34	7.70	0.0	111	56	66.47	-0.40	-0.4200	-0.2774	0.0000	2.4556	-0.8200	66.47	-0.8200	1.7782	66.47
22	5	34	7.70	0.0	60	73	45.11	-0.40	-0.4200	-0.2774	0.0000	2.4556	-0.8200	45.11	-0.8200	1.7782	45.11
22	6	34	7.70	0.0	96	41	70.07	-0.40	-0.4200	-0.2774	0.0000	2.4556	-0.8200	70.07	-0.8200	1.7782	70.07
14	1	34	7.70	2.2	80	75	51.61	-0.40	-0.2667	0.0000	0.0000	2.4556	-0.6667	51.61	-0.6667	2.0556	51.61
14	2	34	7.70	2.2	94	68	58.02	-0.40	-0.2667	0.0000	0.0000	2.4556	-0.6667	58.02	-0.6667	2.0556	58.02
14	3	34	7.70	2.2	92	66	58.23	-0.40	-0.2667	0.0000	0.0000	2.4556	-0.6667	58.23	-0.6667	2.0556	58.23
14	4	34	7.70	2.2	101	57	63.92	-0.40	-0.2667	0.0000	0.0000	2.4556	-0.6667	63.92	-0.6667	2.0556	63.92
14	5	34	7.70	2.2	79	57	58.09	-0.40	-0.2667	0.0000	0.0000	2.4556	-0.6667	58.09	-0.6667	2.0556	58.09
14	6	34	7.70	2.2	109	65	62.64	-0.40	-0.2667	0.0000	0.0000	2.4556	-0.6667	62.64	-0.6667	2.0556	62.64
6	1	34	7.70	6.3	126	43	74.56	-0.40	0.0000	0.4825	0.0000	2.4556	-0.4000	74.56	-0.4000	2.5382	74.56
6	2	34	7.70	6.3	99	76	56.57	-0.40	0.0000	0.4825	0.0000	2.4556	-0.4000	56.57	-0.4000	2.5382	56.57
6	3	34	7.70	6.3	87	100	46.52	-0.40	0.0000	0.4825	0.0000	2.4556	-0.4000	46.52	-0.4000	2.5382	46.52
6	4	34	7.70	6.3	96	80	54.55	-0.40	0.0000	0.4825	0.0000	2.4556	-0.4000	54.55	-0.4000	2.5382	54.55
6	5	34	7.70	6.3	87	58	60.00	-0.40	0.0000	0.4825	0.0000	2.4556	-0.4000	60.00	-0.4000	2.5382	60.00
6	6	34	7.70	6.3	67	72	48.20	-0.40	0.0000	0.4825	0.0000	2.4556	-0.4000	48.20	-0.4000	2.5382	48.20

SUPPLEMENTARY MATERIAL: Fertilization success and multiple stressors

plate	rep	S	pH	temp	fert	unfert	% fert	stress unit pH	stress unit temp 6.3	stress unit temp 2.2	stress unit salinity 6.3	stress unit salinity (2.2°C)	Total stress	% fert	Total stress 3 (temp 6.3)	Total stress 3 (temp 2.2)	% fert
17	1	34	8.10	0.0	106	77	57.92	0.00	-0.4200	-0.2774	0.0000	2.4183	-0.4200	57.92	-0.4200	2.1409	57.92
17	2	34	8.10	0.0	87	102	46.03	0.00	-0.4200	-0.2774	0.0000	2.4183	-0.4200	46.03	-0.4200	2.1409	46.03
17	3	34	8.10	0.0	96	94	50.53	0.00	-0.4200	-0.2774	0.0000	2.4183	-0.4200	50.53	-0.4200	2.1409	50.53
17	4	34	8.10	0.0	118	51	69.82	0.00	-0.4200	-0.2774	0.0000	2.4183	-0.4200	69.82	-0.4200	2.1409	69.82
17	5	34	8.10	0.0	143	19	88.27	0.00	-0.4200	-0.2774	0.0000	2.4183	-0.4200	88.27	-0.4200	2.1409	88.27
17	6	34	8.10	0.0	118	44	72.84	0.00	-0.4200	-0.2774	0.0000	2.4183	-0.4200	72.84	-0.4200	2.1409	72.84
21	1	34	8.10	0.0	226	145	60.92	0.00	-0.4200	-0.2774	0.0000	2.4183	-0.4200	60.92	-0.4200	2.1409	60.92
21	2	34	8.10	0.0	67	110	37.85	0.00	-0.4200	-0.2774	0.0000	2.4183	-0.4200	37.85	-0.4200	2.1409	37.85
21	3	34	8.10	0.0	94	53	63.95	0.00	-0.4200	-0.2774	0.0000	2.4183	-0.4200	63.95	-0.4200	2.1409	63.95
21	4	34	8.10	0.0	69	75	47.92	0.00	-0.4200	-0.2774	0.0000	2.4183	-0.4200	47.92	-0.4200	2.1409	47.92
21	5	34	8.10	0.0	121	51	70.35	0.00	-0.4200	-0.2774	0.0000	2.4183	-0.4200	70.35	-0.4200	2.1409	70.35
21	6	34	8.10	0.0	121	46	72.46	0.00	-0.4200	-0.2774	0.0000	2.4183	-0.4200	72.46	-0.4200	2.1409	72.46
9	1	34	8.10	2.2	109	80	57.67	0.00	-0.2667	0.0000	0.0000	2.4183	-0.2667	57.67	-0.2667	2.4183	57.67
9	2	34	8.10	2.2	109	57	65.66	0.00	-0.2667	0.0000	0.0000	2.4183	-0.2667	65.66	-0.2667	2.4183	65.66
9	3	34	8.10	2.2	102	45	69.39	0.00	-0.2667	0.0000	0.0000	2.4183	-0.2667	69.39	-0.2667	2.4183	69.39
9	4	34	8.10	2.2	105	29	78.36	0.00	-0.2667	0.0000	0.0000	2.4183	-0.2667	78.36	-0.2667	2.4183	78.36
9	5	34	8.10	2.2	113	65	63.48	0.00	-0.2667	0.0000	0.0000	2.4183	-0.2667	63.48	-0.2667	2.4183	63.48
9	6	34	8.10	2.2	74	19	79.57	0.00	-0.2667	0.0000	0.0000	2.4183	-0.2667	79.57	-0.2667	2.4183	79.57
13	1	34	8.10	2.2	90	72	55.56	0.00	-0.2667	0.0000	0.0000	2.4183	-0.2667	55.56	-0.2667	2.4183	55.56
13	2	34	8.10	2.2	97	69	58.43	0.00	-0.2667	0.0000	0.0000	2.4183	-0.2667	58.43	-0.2667	2.4183	58.43
13	3	34	8.10	2.2	90	72	55.56	0.00	-0.2667	0.0000	0.0000	2.4183	-0.2667	55.56	-0.2667	2.4183	55.56

SUPPLEMENTARY MATERIAL: Fertilization success and multiple stressors

plate	rep	S	pH	temp	fert	unfert	% fert	stress unit pH	stress unit temp 6.3	stress unit temp 2.2	stress unit salinity 6.3	stress unit salinity (2.2°C)	Total stress	% fert	Total stress 3 (temp 6.3)	Total stress 3 (temp 2.2)	% fert
13	4	34	8.10	2.2	91	87	51.12	0.00	-0.2667	0.0000	0.0000	2.4183	-0.2667	51.12	-0.2667	2.4183	51.12
13	5	34	8.10	2.2	103	71	59.20	0.00	-0.2667	0.0000	0.0000	2.4183	-0.2667	59.20	-0.2667	2.4183	59.20
13	6	34	8.10	2.2	101	65	60.84	0.00	-0.2667	0.0000	0.0000	2.4183	-0.2667	60.84	-0.2667	2.4183	60.84
1	1	34	8.10	6.3	128	24	84.21	0.00	0.0000	0.4825	0.0000	2.4183	0.0000	84.21	0.0000	2.9008	84.21
1	2	34	8.10	6.3	155	27	85.16	0.00	0.0000	0.4825	0.0000	2.4183	0.0000	85.16	0.0000	2.9008	85.16
1	3	34	8.10	6.3	122	22	84.72	0.00	0.0000	0.4825	0.0000	2.4183	0.0000	84.72	0.0000	2.9008	84.72
1	4	34	8.10	6.3	149	22	87.13	0.00	0.0000	0.4825	0.0000	2.4183	0.0000	87.13	0.0000	2.9008	87.13
1	5	34	8.10	6.3	119	36	76.77	0.00	0.0000	0.4825	0.0000	2.4183	0.0000	76.77	0.0000	2.9008	76.77
1	6	34	8.10	6.3	127	48	72.57	0.00	0.0000	0.4825	0.0000	2.4183	0.0000	72.57	0.0000	2.9008	72.57
5	1	34	8.10	6.3	142	26	84.52	0.00	0.0000	0.4825	0.0000	2.4183	0.0000	84.52	0.0000	2.9008	84.52
5	2	34	8.10	6.3	124	53	70.06	0.00	0.0000	0.4825	0.0000	2.4183	0.0000	70.06	0.0000	2.9008	70.06
5	3	34	8.10	6.3	151	44	77.44	0.00	0.0000	0.4825	0.0000	2.4183	0.0000	77.44	0.0000	2.9008	77.44
5	4	34	8.10	6.3	145	55	72.50	0.00	0.0000	0.4825	0.0000	2.4183	0.0000	72.50	0.0000	2.9008	72.50
5	5	34	8.10	6.3	112	33	77.24	0.00	0.0000	0.4825	0.0000	2.4183	0.0000	77.24	0.0000	2.9008	77.24
5	6	34	8.10	6.3	121	55	68.75	0.00	0.0000	0.4825	0.0000	2.4183	0.0000	68.75	0.0000	2.9008	68.75
